# Response: Commentary: Can Inner Experience Be Apprehended in High Fidelity? Examining Brain Activation and Experience from Multiple Perspectives

**DOI:** 10.3389/fpsyg.2017.00628

**Published:** 2017-04-25

**Authors:** Russell T. Hurlburt, Ben Alderson-Day, Charles Fernyhough, Simone Kühn

**Affiliations:** ^1^Department of Psychology, University of Nevada, Las VegasLas Vegas, NV, USA; ^2^Department of Psychology, Durham UniversityDurham, UK; ^3^Center for Lifespan Psychology, Max Planck Institute for Human DevelopmentBerlin, Germany; ^4^Department of Psychiatry and Psychotherapy, University Clinic Hamburg-EppendorfHamburg, Germany

**Keywords:** descriptive experience sampling, pristine inner experience, mentalism, subjective experience, self-observation, introspection

Schlinger (2017) provided a critique of Hurlburt et al. (2017). We thank him for setting the occasion for important clarifications of pristine inner experience and methods used to apprehend it.

## Pristine experience is not a mentalism, not A mental state, not A cognitive state, not A process

When Skinner (1977) wrote that “The appeal to cognitive states and processes is a diversion which could well be responsible for much of our failure to solve our problems” (p. 10), he meant that human problems are behavioral and that focusing on mental states is an obstacle to understanding and changing our behavior (Schlinger, 2017, paragraph 8).

We entirely agree with Skinner, and have said so repeatedly (e.g., Hurlburt and Heavey, 2001; Hurlburt, 2011a). However, Schlinger is mistaken to include pristine inner experience in the mentalism bucket.

A mentalism is an inner psychological event that is not seen, heard, tasted, or otherwise observed, but is nonetheless assumed to exist (by inference, from theory) and believed to explain behavior. Cognitive states, mental states, hunger, concepts, associations, ideas, identity, will, preference, intention, cognitive rules, encoding, storage, retrieval, knowledge, propositions, representation are all mentalisms (Skinner, 1977). One of the defining aspects of Skinner's career is his polemic against the admission of mentalisms into science: “We must remember that mentalistic explanations explain nothing” (Skinner, 1974, p. 224).

We entirely agree with Skinner's criticism of mentalism (Hurlburt and Heavey, 2001). In fact, it can be fairly said that descriptive experience sampling, and its subject matter pristine inner experience, is explicitly an attempt to describe characteristics of inner experience that are *not* mentalistic. Mentalisms are inferred or theorized states or functions; pristine inner experience is directly apprehended (albeit privately). We claim that Susan at 1:52:12 was directly apprehending (imaginarily) her boyfriend (his mother, too); she was not *inferring* that the boyfriend was present to her; she was not *theorizing about* the boyfriend's presence-to-her. We did *not* claim that Susan was in some *mental* or *cognitive state* (that putatively resulted in the seeing of the boyfriend)—we know nothing about any state. We claimed direct apprehension: Susan was innerly *seeing* the boyfriend.

Mentalisms are advanced as causative explanations. We have never claimed any causal significance for Susan's innerly seeing (nor for any other pristine experience), neither that something caused Susan's inner seeing nor that her inner seeing caused anything. We have claimed only that Susan apprehended her inner experience. We have not *denied* causative significance of pristine inner experience; instead, we have bracketed it away—we have repeatedly stated that our DES task is limited to describing the phenomena that present themselves, and that “those who describe phenomena [should be] firewalled away from those who theorize about the significance of those phenomena” (Hurlburt and Akhter, 2008, p. 1,372; cf, Hurlburt, 2011a, ch. 2, 15).

## “Private” does not necessarily imply non-observable

Susan's inner seeing of her boyfriend is a private event—such a seeing could not possibly present itself to anyone other than Susan. Privacy of observation is indeed a problem for science, but it is not the terminal problem that Schlinger maintains. Our claim is that Susan's inner experience, while private, was directly apprehended.

We cited Hurlburt's “claim that pristine inner experience is …directly apprehendable, as Skinner and the behaviorists required” (Hurlburt et al., 2017, paragraph 15). Schlinger responded that “this statement misrepresents not only what behaviorists require, but what all scientists require: that phenomena be observable” (Schlinger, 2017, paragraph 3). However, that is not what Skinner required, as Schlinger himself noted in 2009:

Non-behavior analysts …believe that behavior analysts only deal with observable behavior. However, contrary to the common depiction of radical behaviorism by cognitively oriented psychologists as denying the existence of private events, behavior analysts explicitly acknowledge their existence and role in the behavioral stream (Skinner, 1945, 1953, 1957). (Schlinger, 2009, pp 79–80)

Hurlburt and Heavey (2001) provided a more complete discussion, leading us to believe that Skinner held (as do we) that the privacy of inner experience makes the science of inner experience difficult but not impossible; Hurlburt (2011a) is an exposition of how to explore inner experience within the constraints that those difficulties require.

Privacy in the study of inner experience is no more fatal than privacy of the physicist alone in her lab reading her voltmeter: the observation is private, and physics must figure out a way (replication, etc.) to validate and integrate the private physicist's result into the network of physical observations. So too must a mature science of inner experience figure out a way to validate and integrate private inner experiences into the network of the experiential. If, for example, it could be shown that inner experience *X* is described by all people who are in situation *Y*, then science could come to accept that *X* is a faithful description of experience, even though all *X* is private. (What significance science should give to *X* is another thing altogether).

## “Private” is not the same thing as “subjective”

The literature uses the term “subjective” in a variety of ways that might, broadly speaking, be divided into three meanings: “private,” “open to bias,” and “loosely specified” (based on no specific referent, casual, impressionistic). Pristine inner experience is, of course, subjective in the “private” usage. Hurlburt (2011a, chapter 17) argued that pristine inner experience might be called “radically non-subjective” to distinguish DES-produced descriptions of pristine experience from the other two usages. “I subjectively feel that crime is rising” refers to no particular crime rate statistics and to no particular personal event (inner or outer). “I rate my anxiety a 7 on a 10 point scale” refers to no shared meaning of 7 or 10, no careful understanding of the nature of or the phenomena of anxiety. By contrast, when Susan says “At 1:52:12 I was seeing my boyfriend in my imagination,” there is a specific referent to her statement—it is either true or false that at that moment she was innerly seeing the boyfriend. It would substantially mischaracterize Susan for her to say, “I subjectively feel that at 1:52:12 I was seeing my boyfriend in my imagination.” She did not *feel* that she (innerly) saw him; she (innerly) *saw* him. The difference between descriptions of pristine inner experience and the other procedures that are typically called “subjective” is important; it can be debated whether “radically non-subjective” was a felicitous descriptor.

## Descriptions of pristine inner experience are not metaphors

Schlinger holds that “descriptions of inner experience are metaphors because when we say that we covertly (or ‘innerly’) ‘see’ or ‘hear’, we are not reacting to actual visual and auditory stimuli” (Schlinger, 2017, paragraph 3). We accept that all language is to some degree metaphorical (Caracciolo and Hurlburt, 2016), but Schlinger's point goes well beyond that, and is, we believe, mistaken.

In the real world, we recognize ourselves as seeing or hearing *not* because of the presence of actual visual or auditory stimuli; we recognize ourselves as *seeing* or as *hearing* because seeing and hearing have distinctly different phenomenal characteristics, and we have lots of experience in the verbal community discriminating between what is seen and what is heard. Suppose you are on the telephone with your trusted friend Chimera. Chimera says, “Hark! A trumpet!” You: “Ah! Do you see a trumpet, or do you hear a trumpet, or both?” Chimera: “I see it.” You: “How do you know you are seeing it, not hearing it?” Would Chimera say, “Because light waves fell on my retinas rather than pressure waves falling on my ear drums”? No; she would say “Because there is a distinct experiential difference between seeing and hearing, a discrimination that has been built up over years of trials in the real world and the verbal community!” Would you believe her? Or would you require that she make objective light-wave measurements? Probably you would trust her discrimination of seeing from hearing because she has in the past made frequent correct discriminations in the real world of trumpets, hillsides, automobiles. If you did suspect that you misunderstood each other (perhaps the telephone connection was breaking up), you could inquire about the trumpet's putatively visual characteristics (Gold or silver? Was someone holding it or was it on a stand? Shiny or dented?) or its auditory characteristics (High or low notes? Loud or soft? Quick or slow?); if she answered appropriately, it would boost your confidence. Chimera's trumpet observation is private, but because Chimera reliably, confidently, mostly unerringly knows the difference between seeing and hearing in the real world, you will likely accept her report as being descriptive, not metaphorical.

In 2009, Schlinger wrote that “behavior analysts assume a behavioral continuity in space and time in which behavior (and stimuli) are on a continuum from public to private (replacing the old distinction between objective and subjective). The only difference is the methodological difficulty of observing private events” (Schlinger, 2009, p. 80). On that logic, the same discriminations that have been acquired in the real world would be expected to apply to inner experience. Susan says she “sees” the boyfriend on the hillside for the same reason that Chimera says she “sees” the trumpet: because the phenomena that are called “seeing” are different from the phenomena that are called “hearing,” or “tasting” or whatever else. Susan's use of “seeing” is no more metaphorical than is Chimera's: both have had their usages refined by the verbal community; both apply their language in private situations; both can be questioned about the relevant details.

## “Apprehend” vs. “observe” vs. “conscious of” vs. “aware of”

Schlinger is correct in writing “when we say that we covertly (or ‘innerly’) ‘see’ or ‘hear,’ we are not reacting to actual visual and auditory stimuli” (Schlinger, 2017, paragraph 3). We are entirely agnostic about whether at 1:52:12 one part of Susan's cognitive or psychic structure creates an image of the boyfriend and another part of Susan's structure sees it; or whether the seeing and the imagined boyfriend are parts of one process. We have therefore avoided such locutions as “Susan is seeing an image of her boyfriend,” because that implies separation between the seer and the thing seen. Instead, we have written “Susan is innerly seeing her boyfriend,” which describes the phenomenon and explicitly leaves the underlying processes unspecified.

For similar reasons, we have avoided using terms like “observe” and “introspect” to describe the DES process, preferring instead “apprehend.” To say that “Susan *observed* her experience that was ongoing at 1:52:12” implies that the experience exists separate from Susan, a claim that we do *not* wish to make. To “observe” connotes remoteness, separation, non-interference, characteristics we have no warrant to presume. Instead, we say that “Susan *apprehended* her experience that was ongoing at 1:52:12.” To “apprehend” connotes proximity, involvement, intervention. We happily accept that the DES process does indeed interfere with Susan's experience, and therefore that her DES-apprehended experience is not in fact pristine—that was our intention when we used *aspired* in “we aspired to faithful apprehensions/descriptions of phenomena as they present themselves of themselves (Hurlburt, 2011a), not skewed or distorted” (Hurlburt et al., 2017, paragraph 27). DES *aspires* to apprehend pristine experience, but we have repeatedly and consistently acknowledged that it always falls short (Hurlburt and Akhter, 2006; Hurlburt and Schwitzgebel, 2007; Heavey et al., 2012; Hurlburt, 2011a,b,c, 2017). The question is the degree to which it falls short, and whether that degree can be controlled by appropriate methods (Hurlburt, 2011a).

So we adopt an agnostic stance toward whether at 1:52:12 Susan is “reacting to actual (or, for that matter, imaginary) visual stimuli.” But we are *not* agnostic about whether at 1:52:12 Susan apprehended herself as innerly seeing her boyfriend. Of course Susan may have lied to us; of course it is possible that the beep caused Susan to *create on the spot* a seeing of her boyfriend and then “recall” it as if it had been ongoing (as Carruthers, 1996, might say, but see Hurlburt and Akhter, 2008; Hurlburt, 2011a for a response).

## Constrained vs. unconstrained explorations of pristine inner experience

Hurlburt et al. (2013), using DES, held that inner speaking—the experience of talking in one's own voice with no external sound and minimal or no muscle movement—is a frequent but by no means ubiquitous phenomenon, and that there are large individual differences in the frequency of inner speaking, ranging from near zero to nearly 100%. Schlinger responds:

Hurlburt et al. …state that some individuals either rarely or never talk to themselves; this is their “pristine experience.” Just because some individuals report rarely talking to themselves does not mean that is so. A simpler explanation is that they are not aware of doing so. In other words, some people may never have learned to label their covert self-talk. (Schlinger, 2017, paragraph 7)

Note that Schlinger does *not* seem to be saying that self-talk is “unconscious” or a “cognitive process” that takes place without awareness; if that were his meaning, then he might have written “A simpler explanation is that *no one* is aware of their self-talk.” Nor does he seem to be saying that self-talk is irrelevant, in which case he might have written, “A simpler explanation is that whether or not people are aware of their self-talk is immaterial to understanding persons because pristine experience is epiphenomenal.” Schlinger wrote neither of those things, nor anything remotely similar, so we conclude that Schlinger is making a claim about pristine inner experience: that (nearly) everyone's pristine inner experience includes frequent self-talk, whether they are aware of it or not.

Thus, Schlinger seems to be claiming that there are two kinds of people: (a) those who engage in self-talk and are aware of it (can therefore label it as such); and (b) those who engage in self-talk but are not aware of it (that is, have never learned to label it correctly).

We think there are actually at least four categories; we would add (c) those who *do not* engage in self-talk and are aware of that lack (and would label self-talk as such if it occurred); and (d) those who *do not* engage in self-talk but mistakenly believe that they do so (that is, have incorrectly labeled non-self-talk experience as being self-talk).

Our DES investigations of inner experience have accepted the possibility of any of those categories, as well as any combination thereof, or any in-between categorization, or any other categorization not yet contemplated. We have not pre-judged, pre-favored, or pre-supposed any results. We have tried to constrain ourselves to discovering the phenomena that present themselves, whatever those phenomena are, taking seriously the hundred or so constraints that Hurlburt (2011a) presented as affecting the reliable apprehensions and characterizations of inner experience. We have worked assiduously to help participants describe (learn to label) inchoate experience, whatever that experience might be; we have also worked assiduously to help participants learn not to mischaracterize phenomena. We have not preferred either kind of descriptive adjustment, in the belief that getting it right (or close thereto) was more important than matching our theories: once experiential phenomena have been reliably characterized, theoretical science (not us!) can determine what (if anything) to make of them.

Across many (we think even-handed) DES investigations over many years, category (d) is far more common than is category (b).

To the extent that Schlinger's disagreement is based on *theoretical* grounds (that self-talk *must* occur and therefore investigations of pristine experience are irrelevant), we have no quarrel (although our crystal ball suggests that that is not a productive course for science). But to the extent that Schlinger's disagreement is based on *observation* of self-talk, we must strongly object, unless he can show that those observations submit to the constraints that Hurlburt (2011a) discussed or are equal or superior in some other way. Casual or armchair introspection is not good enough (Hurlburt and Schwitzgebel, 2007, 2011)—there are far too many opportunities for publicly or privately held theory to bias, perhaps with huge effects, reports about inner experience unless adequate constraints are in place.

We happily accept the criticism that even though we and DES *try* to submit to the constraints Hurlburt (2011a) outlines, there is no guarantee that that submission is successful. That is indeed an important issue for science to grapple with, and Hurlburt et al. (2017) is one small contribution. We happily accept that it is a defensible position in 2017 to hold that science should exclude *all* first-person observation. But we think it is *not* adequate to substitute one's own unconstrained (casual or armchair) self-observation for the DES carefully crafted attempts at apprehension.

## In sum

Pristine inner experiences are directly apprehended phenomena, not mentalisms, not the result of inference or theory. There is reason to believe (although the jury is still out) that there is scientific utility in understanding pristine inner experience.

Descriptive experience sampling (DES) has a limited goal: to apprehend and describe with fidelity ongoing pristine inner experience. It does not seek to specify any causative significance for pristine experience—that is left to others. DES is a careful method, trying to live within the constraints discussed by Hurlburt (2011a) as it describes phenomena as they present themselves.

We are critical of unconstrained attempts at self-observation.

## Author contributions

RH drafted this response on behalf of the other original authors. BA, CF, and SK consulted in the process.

### Conflict of interest statement

The authors declare that the research was conducted in the absence of any commercial or financial relationships that could be construed as a potential conflict of interest.
